# Pharmacokinetics-Pharmacodynamics Analysis of Bicyclic 4-Nitroimidazole Analogs in a Murine Model of Tuberculosis

**DOI:** 10.1371/journal.pone.0105222

**Published:** 2014-08-20

**Authors:** Suresh B. Lakshminarayana, Helena I. M. Boshoff, Joseph Cherian, Sindhu Ravindran, Anne Goh, Jan Jiricek, Mahesh Nanjundappa, Amit Nayyar, Meera Gurumurthy, Ramandeep Singh, Thomas Dick, Francesca Blasco, Clifton E. Barry, Paul C. Ho, Ujjini H. Manjunatha

**Affiliations:** 1 Novartis Institute for Tropical Diseases, Singapore, Singapore; 2 Tuberculosis Research Section, Laboratory of Clinical Infectious Diseases, National Institute of Allergy and Infectious Diseases, National Institutes of Health, Bethesda, Maryland, United States of America; 3 Department of Pharmacy, National University of Singapore, Singapore, Singapore; University of KwaZulu-Natal, South Africa

## Abstract

PA-824 is a bicyclic 4-nitroimidazole, currently in phase II clinical trials for the treatment of tuberculosis. Dose fractionation pharmacokinetic-pharmacodynamic studies in mice indicated that the driver of PA-824 *in* *vivo* efficacy is the time during which the free drug concentrations in plasma are above the MIC (*fT_>MIC_*). In this study, a panel of closely related potent bicyclic 4-nitroimidazoles was profiled in both *in* *vivo* PK and efficacy studies. In an established murine TB model, the efficacy of diverse nitroimidazole analogs ranged between 0.5 and 2.3 log CFU reduction compared to untreated controls. Further, a retrospective analysis was performed for a set of seven nitroimidazole analogs to identify the PK parameters that correlate with *in* *vivo* efficacy. Our findings show that the *in* *vivo* efficacy of bicyclic 4-nitroimidazoles correlated better with lung PK than with plasma PK. Further, nitroimidazole analogs with moderate-to-high volume of distribution and Lung to plasma ratios of >2 showed good efficacy. Among all the PK-PD indices, total lung *T_>MIC_* correlated the best with *in* *vivo* efficacy (*r_s_* = 0.88) followed by lung C_max_/MIC and AUC/MIC. Thus, lung drug distribution studies could potentially be exploited to guide the selection of compounds for efficacy studies, thereby accelerating the drug discovery efforts in finding new nitroimidazole analogs.

## Introduction

Every year nearly 8 million new cases of Tuberculosis (TB) are reported globally resulting in 1.4 million deaths [1]. Poor treatment compliance – due to the requirement for prolonged multidrug therapy – as well as the use of inadequate regimens has fueled the emergence of multi-drug-resistant and extensively-drug-resistant (MTD-TB and XDR-TB) TB strains. MDR-TB is resistant to at least isoniazid and rifampicin and XDR-TB is resistant to isoniazid, rifampicin, fluoroquinolones and at least one of the injectables [2]. TB control programs are further complicated in settings where the incidence of co-infection with HIV is high because drug-drug interactions with anti-retroviral therapy are difficult to avoid [2], [3]. Hence there is an urgent need to discover new TB drugs active against drug-resistant forms of TB and compatible with treatment against HIV.

PA-824 [4] and OPC-67683 [5] are two bicyclic 4-nitroimidazoles currently in phase II clinical trials, representing a promising new class of therapeutics for TB [6]. Preclinical testing of PA-824 showed bactericidal activity in various *in* *vitro* and *in* *vivo* models [7], . PA-824 was shown to be well tolerated in healthy subjects, following oral daily doses for 7 days [9]. These results, combined with the demonstrated activity of PA-824 against drug-sensitive and multidrug-resistant Mtb, supported the progression of this compound and its evaluation as a novel treatment against TB. An early bactericidal activity (EBA) study of PA-824 revealed a lack of dose response between 200 and 1200 mg administered daily for 14 days [10]. Dose-fractionation PK-PD studies in mice showed the PK-PD driver of PA-824 to be the time during which the free drug concentrations in plasma were above the MIC (*fT_>MIC_*) [11]. In retrospect, clinical investigators established that *fT_>MIC_* was 100% at all doses between 200 and 1200 mg daily. An additional phase II trial between 50 and 200 mg was undertaken to establish the lowest efficacious dose [12]. 200 mg of PA-824 was found to be efficacious and used in combination with other anti-TB drugs [13].

Physico-chemical properties, *in* *vitro* potency, *in* *vitro* and *in* *vivo* pharmacokinetics (PK) are critical determinants for *in* *vivo* efficacy. PA-824 is highly lipophilic and exhibits poor aqueous solubility. To overcome the limitation of its low solubility and improve its oral bioavailability, a cyclodextrin formulation was developed and used for *in* *vivo* animal efficacy studies [4], [7]. Extensive lead optimization efforts were undertaken to improve aqueous solubility, metabolic stability, *in* *vitro* potency and *in* *vivo* efficacy of anti-tubercular nitroimidazoles and various analogs were synthesized [5], [14]–[23]. Comprehensive *in* *vivo* pharmacology studies are generally resource and time intensive. This is particularly true for TB because of the slow rate of *M. tuberculosis* growth, lengthy treatment duration and requirement of high containment facility. In this study, a panel of closely related potent bicyclic 4-nitroimidazoles (NI) was profiled both *in* *vitro* and *in* *vivo*. The data is retrospectively analyzed to identify the PK parameters that correlate with *in* *vivo* efficacy of a series of bicyclic 4-nitroimidazoles. The results of this investigation showed that PK properties such as volume of distribution and lung exposure predicts *in* *vivo* efficacy of bicyclic 4-nitroimidazoes better than other PK parameters. Thus, *in* *vitro* potency and lung PK could be used as surrogate to guide the prioritization of new pre-clinical candidates for lengthy efficacy studies, there by expediting drug discovery.

## Materials and Methods

### Bacteria, culture conditions and chemicals

*M. tuberculosis* (Mtb) (H37Rv, ATCC 27294) culture conditions have been described previously [16]. Synthesis of PA-824, Amino-824, Aminoethyl-824, NI-135, NI-147 and NI-136 have been previously reported [16], [20]. Other NI analogs NI-622, NI-644, NI-176, NI-269, NI-182, NI-145, NI-297 and NI-302 have been described in two patents [14], [18] and synthesis of these compounds to be described elsewhere. All solutions were made as 20 mM stocks in DMSO. Hydroxypropyl-β-cyclodextrin, Lecithin, granular was purchased from Acros/Organics, New Jersey, and USA. Minimum Inhibitory concentration (MIC_99_) against wild type Mtb H37Rv and cofactor F_420_ deficient (*FbiC* mutant) [24] was determined by the broth dilution method as described earlier [16].

### *In vitro* physico-chemical properties

*In vitro* physicochemical and PK parameters like solubility, log P, PAMPA, Caco-2 permeability and mouse plasma protein binding were determined in-house in medium to high throughput format assays. Briefly, solubility was measured using a high throughput equilibrium-solubility (HT-Eq sol) assay using a novel miniaturized shake-flask approach and streamlined HPLC analysis [25]; lipophilicity determination was carried out in 96-well micro titer plates and the diffusion of compounds between two aqueous compartments separated by a thin octanol liquid layer was measured [26]; PAMPA permeability experiments were carried out in 96-well micro titer filter plates at absorption wavelengths between 260 and 290 nm [27]; Caco-2 assay was carried out in a 96-well format, and compound concentrations in each chamber were measured by LC/MS as described previously [28] and plasma protein binding was determined in mouse plasma using an ultra-filtration method [29].

### Ethics Statement

All animal experimental protocols (protocol #023/2009 and #025/2009 for PK; protocol #004/2010 for efficacy) involving mice were reviewed and approved by the Institutional Animal Care and Use Committee (IACUC) of Novartis Institute for Tropical Diseases (NITD). The animal research complied with Singapore Animal Veterinary Authority and global Novartis policy on the care and use of animals. Experimental and control animals infected with Mtb were euthanized at the end of the experiment. All procedures during pharmacokinetic experiments were performed under isoflurane anesthesia and all efforts were made to minimize suffering.

### *In vivo* pharmacokinetic (PK) studies

Female CD-1 mice obtained from Biological Resource Center in Singapore were used for *in* *vivo* PK studies. Mice were acclimatized before initiation of pharmacokinetic (PK) experiments. Feed and water were given *ad libitum*. The compounds were formulated at a concentration of 1, 2.5 or 5 mg/mL for a dose of 10 mg/kg (Amino-824, AminoEthyl-824) or 25 mg/kg (PA-824, NI-135, NI-147, NI-136, NI-176, NI-269, NI-182, NI-145, NI-297 and NI-302) or 50 mg/kg (NI-622 and NI-644) given orally and at 1 or 2 mg/mL concentration for a dose of 5 or 10 mg/kg given intravenously. The CM-2 formulations were prepared in 10% w/v hydroxypropyl-β-cyclodextrin and 10% v/v lecithin in water as described earlier [7], [8]. The formulation was centrifuged and the supernatant was collected for intravenous administration. After oral dosing, blood and lung samples from mice were collected at various time points ranging from 0.08 hrs (but 0.02 hrs for i.v dosing) to 48 hrs. Groups of three mice were used for each time point. Blood was centrifuged at 13,000 rpm for 7 min at 4°C, plasma was harvested and stored at –20°C until analysis. Lung tissue samples were excised, dipped in PBS, gently blotted with absorbent paper, dried, weighed and stored at –20°C until further analysis.

For LC/MS/MS analysis, 50 µL of plasma samples were precipitated using 400 µL of acetonitrile:methanol:acetic acid (90∶9.8∶0.2) containing 500 ng/mL of either related compound or warfarin as internal standard. After vortexing and centrifuging the mixture, the supernatant was removed and 5 µL of sample analyzed. Whole lung tissue was homogenized in 2 mL of PBS. 50 µL of lung homogenate was taken and processed as described above for plasma samples. The standard calibration curve was prepared by spiking blank plasma and lung tissue with different concentrations of the compound. In addition, quality control samples with three different concentrations were prepared in respective blank matrix and analyzed together with the unknown samples for validation purposes. Analyte quantitation was performed by LC/MS/MS using optimized conditions for each compound. Liquid chromatography was performed using an Agilent 1100 HPLC system (Santa Clara, CA), with the Agilent Zorbax XDB Phenyl (3.5 µ, 4.6×75 mm) column at an oven temperature of 35°C and 45°C, coupled with a triple quadruple mass spectrometer (Applied Biosystems, Foster City, CA). Instrument control and data acquisition were performed using Applied Biosystems software Analyst 1.4.2. The mobile phases used were A: water-acetic acid (99.8∶0.2, v/v) and B either as: acetonitrile-acetic acid (99.8∶0.2, v/v) or methanol-acetic acid (99.8∶0.2, v/v), using a gradient, with a flow rate of 1.0 mL/min, and a run time of 6 to 8 min. Under these conditions the retention times of various compounds ranged between 3.2 and 6.5 minutes. Multiple reaction monitoring (MRM) was combined with optimized mass spectrometry parameters to maximize detection specificity and sensitivity. Most of the compounds were analyzed using electrospray ionization in the positive mode. The recovery of the compound from both plasma and lung tissue were good and consistent across the concentration range studied. The lower limit of quantification for different compounds ranged between 1 and 49 ng/mL in plasma and 1 and 132 ng/g in lungs. Calibration curve was prepared freshly and analyzed with every set of study samples. Intraday variability was established with triplicate quality control samples at three concentration levels. The results were accepted if relative standard deviation was less than 15%.

Mean values of compound concentrations in plasma and lungs were obtained from three animals at each time point and plotted against time to generate concentration-time profiles. PK parameters were determined using Phoenix WinNonlin, version 6.3 (Pharsight, A Certara company, USA, www.pharsight.com), by non-compartmental modeling using built-in model (200–202) for extravascular and intravenous bolus dosing. The oral bioavailability (F) was calculated as the ratio between the area under the curve (AUC_inf_) following oral administration and the AUC_inf_ following intravenous administration corrected for dose (F = AUC_p.o_*dose_i.v._/AUC_i.v_*dose_p.o_)_._

### *In vivo* mouse efficacy studies

*In vivo* mouse efficacy studies were determined after intranasal infection of Balb/c mice with 10^3^ cfu Mtb H37Rv. Treatment was initiated 4 weeks after infection. Compounds were orally administered in CM-2 formulation for 4 weeks daily. Bacterial loads were determined at 2 and 4 weeks post treatment [30]. Statistical analysis was done by a one-way analysis of variance, followed by a multiple comparison analysis of variance by a one-way Tukey test (GraphPad Prism, version 5.02, San Diago, California USA, www.graphpad.com). Differences were considered statistically significant at the 95% level of confidence [7].

### Calculation of PK-PD parameters

The MIC against Mtb was used to calculate PK-PD indices (C_max_/MIC, AUC/MIC and *T_>MIC_*). The C_max_/MIC was defined as the ratio of peak plasma concentration (C_max_) to the MIC, the AUC/MIC was defined as the ratio of the AUC_0–24_ to the MIC, and the time above MIC (*T_>MIC_*) was defined as 24 h period during which the total compound concentration exceeded the MIC. C_max_/MIC and AUC/MIC were calculated as ratios from PK parameter obtained from non-compartmental analysis and MIC. *T_>MIC_* were derived from Phoenix WinNonlin software by specifying MIC as therapeutic response and time above therapeutic response was obtained. Using plasma protein binding, unbound concentrations in plasma were calculated, PK parameters were derived from Phoenix WinNonlin and PK-PD indices were defined as *f*C_max_/MIC, *f*AUC/MIC and % *fT_>MIC_* where ‘*f*’ refers to free concentration. For calculation and plotting of mean concentration-time curve, concentrations indicated as below the lower limit of quantification (LLOQ) were replaced by 0.5*LLOQ. Ignoring the values here would impact some of the PK-PD parameters. This approach has no impact on pharmacokinetic parameter calculations [31].

### PK-PD analysis

PK-PD indices were estimated from the plasma and lung drug concentrations, *in* *vitro* potency and plasma protein binding. A Spearman’s rank correlation [32], [33] was run to determine the relationship between various PK parameters and mean log CFU reduction using Prism software (GraphPad Prism, version 5.02, San Diago, California USA, www.graphpad.com).

## Results

### *In vitro* potency and physico-chemical properties

**Figure 1:**
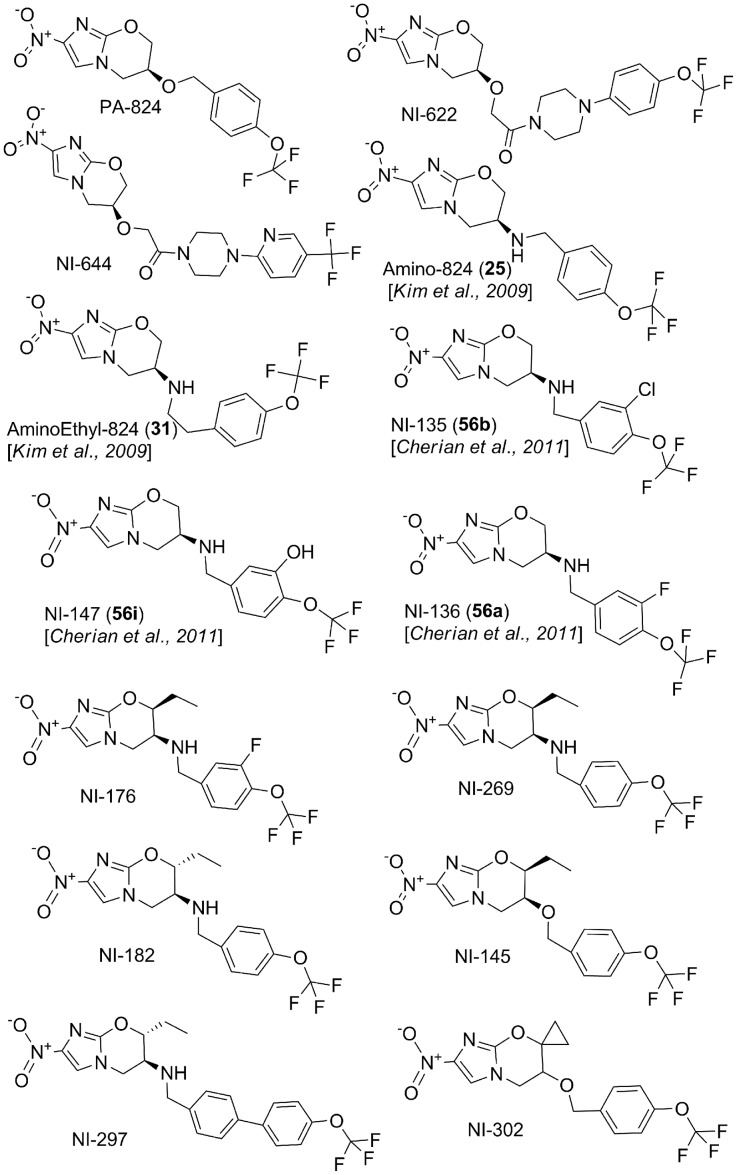
Chemical structures of bicyclic 4-nitroimidazole analogs used in this study.

In an effort to improve the solubility and potency of PA-824, diverse structural analogs of PA-824 were synthesized and their *in* *vitro* activities reported [14], [16], [18]–[20]. A few potent bicyclic 4-nitroimidazole analogs were selected and characterized in detail (Figure 1). *In vitro* Mtb potency and physico-chemical properties of these nitroimidazole analogs are summarized in Table 1. All the NI analogs studied showed Mtb specific growth inhibitory activity and no cytotoxicity was observed in THP1 macrophage cell lines (Table 1). F_420_ deficient (*FbiC*) mutants were resistant to all these bicyclic 4-nitroimidazoles analogs (Table 1), suggesting a mechanism of action similar to PA-824, involving F_420_-dependent bio-activation [24]. Modifications on the benzyl ring (NI-135, NI-147, NI-136 and NI-176), and oxazine ring (NI-269, NI-182, NI-145, NI-297, NI-302 and NI-176) showed significant improvement of *in* *vitro* potency compared to PA-824. The nitroimidazole (NI) analogs tested in this study displayed a wide range of solubility (<2 to 127 µg/mL). Amino-nitroimidazoles showed improved solubility compared to their respective benzyl ether analogs (Amino-824 vs. PA-824 and NI-269 vs. NI-145). NI-297, a biphenyl derivative of NI-182, had very poor aqueous solubility (<2 µg/mL) due to its high lipophilicity (logP 6). In general, the logP of all the other NI derivatives ranged between 2.4 and 3.8 and all showed high permeability except NI-644, which had moderate permeability in the Caco-2 assay. Overall the compounds exhibited moderate-to-high mouse plasma protein binding (80 to 98%), except for Aminoethyl-824, which showed the lowest binding (45%).

**Table 1:** *In vitro* potency and physicochemical properties for bicyclic 4-nitroimidazole analogs.

Compound ID	H37Rv MIC_99_(mg/L)	H37Rv::F_420_-mutantsMIC_99_ (mg/L)	CytotoxicityCC_50_ (mg/L)	SolubilitypH 6.8(µg/mL)	Log P	PAMPA(Log Pecm/sec, %FA)	CaCo-2(Papp, 10^−6^ cm/sec)			CaCo-2(Efflux ratio)	Mice PPB (%)
							A-B		B-A	B-A/A-B	
PA-824	0.30	>10	>10	13.0	2.75	–4.2, 99	27.6		20.4	0.8	90
NI-622	0.18	>10	>10	28.2	2.9	–5.3, 31	9.5		12.4	1.3	97
NI-644	0.09	>10	>10	14.0	2.6	–4.9, 83	2.8		13.7	4.8	90
Amino-824	0.14	>10	>10	127	2.5	–4.8, 91	35.4		21.0	0.6	80
AminoEthyl-824	0.06	>10	>10	104	3.6	–4.6, 85	18.6		20.7	1.1	45
NI-135	0.03	>10	>10	77.0	3	–4.5, 90	23.4		15.3	0.6	91.5
NI-147	0.03	>10	nd	56.0	2.4	–5.2, 43	nd				nd
NI-136	0.03	>10	>10	43.0	2.6	–4.7, 83	15.4		12.6	0.8	82.7
NI-176	0.03	>10	>10	8.0	3.5	–3.7, 99	nd				nd
NI-269	0.03	>10	>10	12.0	3.15	–3.7, 99	nd				nd
NI-182	0.02	>10	>10	32.0	3.05	–3.8, 99	18.3		13.9	0.8	91.3
NI-145	0.06	>10	>10	<2.0	3.85	–3.85, 99	nd				nd
NI-297	0.02	>10	>10	<2.0	6	–4.1, 97	nd				98.2
NI-302	0.03	>10	>10	26.0	3.2	–3.6, 99	45.1	18.7		0.4	95.7

MIC_99_ = Minimum Inhibitory Concentration required to reduce the bacterial growth by 99%, MIC against both H37Rv (wild type) and F_420_ deficient (*FbiC*) mutants were tested. PAMPA = Parallel Artificial Membrane Permeability Assay, CaCo-2 = Permeability using colon carcinoma cell lines, PPB = Plasma Protein Binding, Pe = effective permeability, FA = fraction absorbed, Papp = apparent permeability, A-B = Apical to Basolateral, B-A = Basolateral to Apical, nd = not determined, CC_50_ = Cytotoxicity against THP1 macrophage cell lines was determined as described previously [76], with Puromycin as positive control (CC_50_ = 1.4 mg/L).

### *In vivo* plasma PK properties

Each compound was subjected to intravenous and oral mouse PK in CM-2 formulation. The total compound concentration in mouse plasma was measured and free plasma concentrations were calculated using *in* *vitro* plasma protein binding (Table 1 and 2). NI-147 with a hydroxyl functional group on the benzyl ring, displayed markedly inferior PK properties (Table 2, Figure S1 in File S1). The poor PK is likely due to glucuronidation of the hydroxyl group as suggested by the presence of an extra peak corresponding to +176 Da in the mass spectrometry analysis. Hence NI-147 was not included in *in* *vivo* mouse efficacy studies. The NI analogs displayed a wide range of volume of distribution (Vss = 0.7 to 4.2 L/kg) corresponding to 1.1 to 7 times total body water. NI-135 showed higher Vss (4.2 L/kg), followed by NI-182, NI-297 and NI-269 (2.6 to 3 L/kg). All other analogs showed moderate Vss similar to PA-824, except for NI-644, which showed a low volume of distribution (Vss = 0.7 L/kg). The total systemic clearance was low to moderate (4 to 44 mL/min/kg) corresponding to 5 to 49% of hepatic blood flow. NI-135 and AminoEhtyl-824 showed moderate clearance (41 and 44 mL/min/kg respectively). All other analogs showed clearance similar to PA-824 (10 to 25 mL/min/kg), except for NI-297 and NI-302, which showed very low clearance (5 mL/min/kg). The elimination half-life ranged between 0.7 h and 6.7 h for the NI analogs studied. NI-297, NI-135 and NI-302 showed long half-life (3.7 to 6.7 h). All other analogs showed half-life similar to PA-824 (1.3 to 2.8 h), except for NI-644 and AminoEthyl-824, which showed short half-life (<1 h). Generally, the NI analogs at comparable doses displayed rapid absorption (T_max_, 0.3 to 4 h), except for NI-297, which showed delayed absorption with a T_max_ of 8 h, possibly due to its higher lipophilicity and lower solubility. The peak plasma concentration (C_max_) ranged between 1.2 µg/mL and 12.9 µg/mL and exposure (AUC) ranged between 4.8 µg.h/mL and 144 µg.h/mL (Table 2). NI-135 had significantly lower plasma C_max_, exposure and oral bioavailability compared to PA-824, likely due to its three times higher *in* *vivo* clearance combined with higher volume of distribution. On the contrary, NI-302 and NI-297 had higher systemic plasma exposure mostly due to decreased *in* *vivo* clearance (Table 2, Figure S1 in File S1). At comparable dose, NI-622 and NI-644 showed similar plasma C_max_, exposure and oral bioavailability compared to PA-824. All other NI analogs showed moderate plasma exposure and oral bioavailability (64 to 88%) except for NI-135, NI-297 and NI-302. Despite, NI analogs displayed varied aqueous solubility (<2 to 127 ug/mL), in CM2 formulation all the analogs showed moderate to high oral bioavailability (Table 2). Interestingly, NI-145 and NI-297 had least solubility (<2 µg/mL), in CM-2 formulation both compounds showed good oral bioavailability. The use of CM-2 (lipid coated cyclodextrin complexation) formulation is known to improve solubility and bioavailability [34]. At 25 mg/kg, the free plasma C_max_ and AUC parameters ranged from 0.1–0.8 µg/mL and 0.4–5.1 µg.h/mL respectively. NI-644 showed similar free plasma concentration as PA-824, whereas all other NI analogs showed relatively lower free plasma C_max_ and exposure (Table S1 in File S1).

**Table 2:** *In vivo* pharmacokinetic parameters in plasma for bicyclic 4-nitroimidazole analogs.

CompoundID	Dose(mg/kg)	p.o. PK parameters					i.v. PK parameters^a^		
		C_max_(µg/mL)	AUC_24_(µg.h/mL)	T_max_(hr)	T_1/2_(p.o.)	F (%)	V_ss_(L/kg)	CL(mL/min/kg)	T_1/2_(i.v)
PA-824*	25	6.0	50.9	2	2.7	100	1.6	12.1	1.6
NI-622*	50	14.8	108.4	4	2.1	100	1.8	14.7	1.8
NI-644*	50	16.2	89.5	1	3.6	100	0.7	9.5	0.9
Amino-824*	10	1.7	6.0	0.3	2.0	74	2.3	20.5	2.0
AminoEthyl-824*	10	1.0	2.9	1	1.7	76	2.0	44.0	0.7
NI-135∧	25	1.2	4.8	0.5	2.9	51	4.2	41.0	4.0
NI-147∧	25	0.04	0.02	0.1	0.2	0.3	0.4	70.8	0.3
NI-136∧	25	2.0	10.7	0.5	2.0	64	1.8	25.1	1.3
NI-176∧	25	2.2	13.7	1	4.3	86	1.8	22.2	1.0
NI-269∧	25	2.8	16.0	0.3	3.8	88	2.6	19.7	2.1
NI-182∧	25	3.5	22.5	0.5	2.0	82	3.0	15.2	2.8
NI-145∧	25	1.8	16.2	2	4.2	68	1.7	17.0	2.0
NI-297∧	25	6.0	99.1	8	4.9	100	2.6	5.0	6.7
NI-302∧	25	12.9	144.1	4	4.1	100	1.2	4.3	3.7

a i.v dosing at either 10 mg/kg* or 5 mg/kg∧.

C_max_ = maximum concentration reached in plasma, AUC_24_ = exposure between 0 to 24 h, T_max_ = time to reach maximum concentration, T_1/2_ = half-life, F = oral bioavailability, Vss = volume of distribution at steady state, CL = total systemic clearance.

### *In vivo* lung PK properties

The primary and the most important site of Mtb infection in patients is lung tissue. To understand the effect of structural changes in the NI molecules on lung PK parameters, we measured total compound concentration in mouse lungs (Table 3). The NI analogs at 25 mg/kg dose showed a wide range of values for lung C_max_ (4.2–17.8 µg/g), T_max_ (0.08–2 h) and exposure (18.6–233 µg*h/g). All NI analogs displayed near parallel concentration-time profile in plasma and lung tissue, suggesting a rapid equilibrium between these two tissues. Interestingly, the lung-to-plasma partitioning varied from 0.5 to 4.6 for C_max_ and 0.4 to 3.9 for AUCs across the series in correlation with the observed volume of distribution (Table 3). NI-135, NI-136, NI-182 and NI-297 showed lung partitioning of 2.5 to 4.6 fold, and are comparable to PA-824. NI-622, NI-644, Amino-824 and NI-302 showed lower lung partitioning (<1) compared to PA-824. Although, NI-135 and NI-136 showed higher lung to plasma ratio (3.6 to 4.6), their absolute lung concentrations were 2.5 to 7.5 fold lower than PA-824. On the contrary, NI-302 showed lower lung partitioning, but its absolute concentrations were comparable to PA-824 (Table 3).

**Table 3:** *In vivo* pharmacokinetic parameters in lungs for bicyclic 4-nitroimidazole analogs.

Compound	Dose(mg/kg)	Lung PK parameters				Lung to Plasma ratio	
		C_max_ (µg/g)	AUC_24_ (µg.h/g)	T_max_ (hr)	T_1/2_ (p.o.)	C_max_	AUC_24_
PA-824	25	17.8	139.9	0.3	4.8	3.0	2.7
NI-622	50	10.2	71.1	0.5	1.8	0.7	0.7
NI-644	50	7.5	38.1	1	2.9	0.5	0.4
Amino-824	10	1.4	4.5	0.5	1.3	0.8	0.8
AminoEthyl-824	10	1.6	5.2	0.5	1.2	1.6	1.8
NI-135	25	5.5	18.6	0.5	3.3	4.6	3.9
NI-147	25	0.5	2.6	0.1	-	-	-
NI-136	25	7.2	39.8	0.5	2.5	3.6	3.7
NI-176	25	4.8	30.3	0.5	4.3	2.2	2.2
NI-269	25	5.4	27.5	0.3	2.7	1.9	1.7
NI-182	25	11.4	73.2	0.5	1.9	3.3	3.3
NI-145	25	4.2	34.6	2	4.1	2.3	2.1
NI-297	25	16.3	233.4	8	4.6	2.7	2.4
NI-302	25	9.7	100.3	2	4.1	0.8	0.7

### Established mouse efficacy

Based on the *in* *vitro* potency and the *in* *vitro* and *in* *vivo* PK results, ten compounds were selected for *in* *vivo* mouse efficacy studies with 4 weeks of daily oral treatment. The mean lung CFU reductions compared to untreated controls are summarized in Table 4. The efficacy ranged from 0.5 to 1.56 log at 25 mg/kg and 0.6 to 2.3 log at 100 mg/kg compared to vehicle treated animals. At 25 mg/kg, NI-622 and NI-644 were significantly (P<0.05) less efficacious than PA-824, however other NI analogs (NI-135, NI-136, NI-182 and NI-297) showed comparable efficacy to PA-824. At 100 mg/kg, AminoEthyl-824, NI-135, NI-136 and NI-302 showed comparable efficacy to PA-824, however NI-622, NI-644, Amino-824 and NI-182 were significantly (P<0.05) less efficacious than PA-824. For PA-824, NI-135 and NI-136 a dose dependent increase in efficacy was observed, on the contrary, no dose-dependent increase in bactericidal activity was observed for NI-622, NI-644 and NI-182. However, none of the selected bicyclic 4-nitroimidazole analogs showed significantly better efficacy than PA-824 at respective 25 and 100 mg/kg doses.

**Table 4:** *In vivo* pharmacodynamics of bicyclic 4-nitroimidazole analogs studied in mice.

	Mean log lung CFU ± SEM			ΔMean log lung CFU reduction ± SEM	
Dose (mg/kg)	Vehicle control	25	100	25	100
PA-824	6.07±0.12	4.67±0.37	4.12±0.13	1.40±0.37	1.95±0.13
	6.24±0.02	4.67±0.10	3.93±0.09	1.57±0.10	2.31±0.09
	6.66±0.14	5.20±0.05	4.19±0.11	1.46±0.05	2.47±0.11
				1.48±0.09$	2.30±0.08$
NI-622	6.66±0.14	5.77±0.08	5.85±0.11	0.89±0.08*	0.81±0.11*
NI-644	6.66±0.14	6.18±0.03	6.09±0.07	0.48±0.03*	0.57±0.07*
Amino-824	6.22±0.08	nd	5.14±0.08	nd	1.08±0.08*
AminoEthyl-824	6.22±0.08	nd	4.53±0.07	nd	1.69±0.07^ns^
NI-135	6.24±0.02	4.76±0.07	4.36±0.07	1.48±0.07^ns^	1.88±0.07^ns^
NI-136	6.24±0.02	4.93±0.06	4.18±0.17	1.31±0.06^ns^	2.06±0.17^ns^
NI-182	6.07±0.12	4.84±0.14	4.81±0.18	1.23±0.14^ns^	1.26±0.18*
NI-297	6.07±0.12	4.51±0.11	nd	1.56±0.11^ns^	nd
NI-302	6.62±0.11	nd	4.45±0.22	nd	2.17±0.22^ns^

The mean log lung CFU’s in five independent *in* *vivo* efficacy studies ranged between 6.07 and 6.66 in untreated infected mice.

nd = not determined.

Δ Mean log lung CFU reduction compared to untreated controls. Each data represents mean value **±** SEM from 5 animals.

$ Mean value from three independent experiments (n = 15).

*Significant difference at P<0.05 compared to respective PA-824 doses.

ns No significant difference at P<0.05 compared to respective PA-824 doses.

### Correlation of PK parameters with efficacy

**Figure 2:**
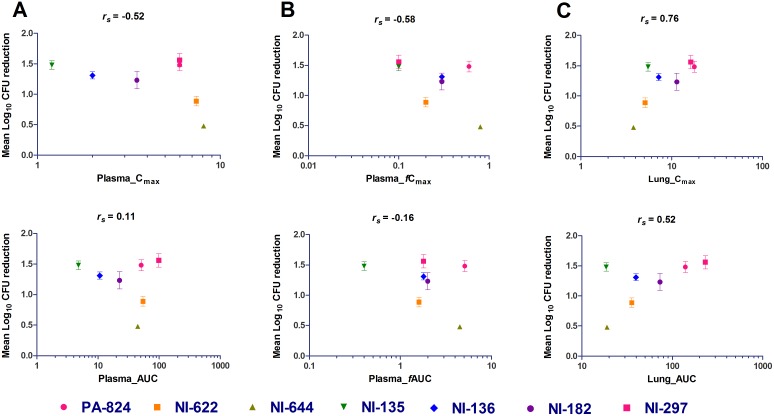
Correlation of PK parameters (C_max_, AUC) with *in vivo* efficacy in mice for bicyclic 4-nitroimidazole analogs in total plasma concentration (A), free plasma concentration (B) and total lung concentration (C). Each data point represents Δ mean log lung CFU reduction compared to untreated controls (mean value ± SEM from 5 animals). *r_s_* is the Spearman’s rank correlations coefficient.

Further, mouse PK and efficacy data was used to understand the relationship of PK parameters with *in* *vivo* efficacy for a series of NI analogs. Both PK and efficacy data at 25 mg/kg were available for only 7 compounds. PK parameters were obtained after a single oral dose (Table 2), while efficacy studies were performed at oral daily doses of 25 and 100 mg/kg for 4 weeks (Table 4). The relationship between mean log CFU reduction with PK parameters (C_max_ and AUC) was analyzed in both plasma and lungs using the Spearman’s rank correlation (Figure 2, Table S1 in File S1). The free plasma concentrations were obtained by correcting for *in* *vitro* mouse plasma protein binding. As shown in Figure 2, with the limited set of compounds, the *in* *vivo* efficacy correlated well with lung PK parameters than plasma PK parameters. The Spearman’s rank correlation coefficient for lung C_max_ and AUC were 0.76 and 0.52 respectively. Across the NI analogs studied, compounds with higher lung concentration (PA-824, NI-297 and NI-182) tended to achieve higher efficacy (Δlog CFUs ranging from 1.23 to 1.56), likewise compounds with lower lung concentration (NI-644 and NI-622) displayed only marginal efficacy (Δlog CFUs ranging from 0.48 to 0.89) (Table 3 and 4). In general, lung C_max_ and exposure showed positive correlation with *in* *vivo* efficacy for bicyclic 4-nitroimidazoles.

### Correlation of PK-PD indices with efficacy

**Figure 3:**
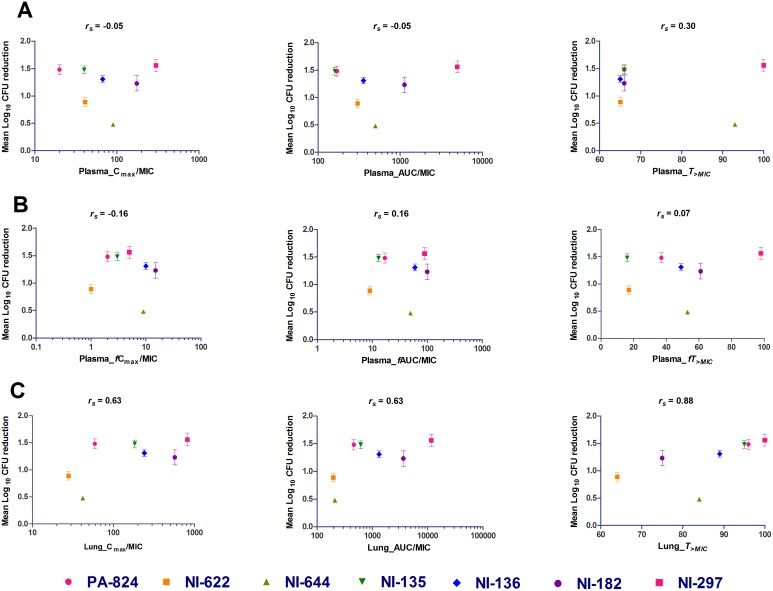
Correlation of PK-PD indices (C_max_/MIC, AUC/MIC and *T_>MIC_*) with *in vivo* efficacy in mice for bicyclic 4-nitroimidazole analogs in total plasma concentration (A), free plasma concentration (B) and total lung concentration (C). Each data point represents Δ Mean log lung CFU reduction compared to untreated controls (mean value ± SEM from 5 animals). *r_s_* is the Spearman’s rank correlations coefficient.

*In vitro* activity against Mtb is one of the key determinants of *in* *vivo* efficacy, hence the relationship between mean log lung CFU reduction with three primary descriptive PK-PD indices (C_max_/MIC, AUC/MIC and *T_>MIC_*) was analyzed in both plasma and lungs (Figure 3, Table S2 in File S1). As observed above, over all, the *in* *vivo* efficacy seems to have strong positive correlation with lung PK parameters than plasma. Among all the PK-PD indices, total lung *T_>MIC_* correlated the best with *in* *vivo* efficacy (*r_s_* = 0.88) than lung C_max_/MIC (*r_s_* = 0.63) and AUC/MIC (*r_s_* = 0.63) (Figure 3C, Table S2 in File S1). For all the compounds analyzed, the total lung *T_>MIC_* ranged between 64 and 100% resulting in 0.9–1.56 log lung CFU reduction. In this analysis, NI-644 was found to be an outlier, with *T_>MIC_* of 84% resulted in only 0.48 log CFU reduction. Overall, these results suggest that *in* *vivo* efficacy of bicyclic 4-nitroimidazole analogs correlates better with the time during which the total lung concentrations are above *in* *vitro* potency.

## Discussion

Understanding pharmacokinetic-pharmacodynamic (PK-PD) relationships in the early drug discovery process is essential to minimize the attrition rate during the pre-clinical and clinical development phases. In murine models of TB, PK-PD relationships have been established for several standard TB drugs, such as rifampicin [35], isoniazid [36], fluoroquinolones (FQ) [37] and TMC207 [38]. Based on the PK-PD findings with rifampicin, further clinical studies are still in progress to optimize the clinical dose [39]–[42]. PA-824, a bicyclic 4-nitroimidazole has demonstrated bactericidal activity in both preclinical and clinical settings [7], [8], [10]. Extensive medicinal chemistry efforts to improve aqueous solubility, metabolic stability, *in* *vitro* potency and *in* *vivo* efficacy have independently generated several series of NI analogs [5], [14]–[23]. All the bicyclic 4-nitroimidazole analogs analyzed in this study showed cofactor F_420_ dependent bio-activation (Table 1) suggesting the mechanism of action of these compounds similar to PA-824 [24], [43]. Comprehensive *in* *vivo* efficacy studies are generally resource/time intensive and are particularly true for TB. Thus, prioritizing potential lead compounds for *in* *vivo* efficacy studies would be useful based on PK parameters. This study is a retrospective analysis of *in* *vivo* efficacy with PK for bicyclic 4-nitroimidazole analogs to identify the PK parameters and PK-PD indices that correlate with the *in* *vivo* potency. The results of this analysis could potentially be exploited to prioritize new analogs for efficacy studies.

**Figure 4:**
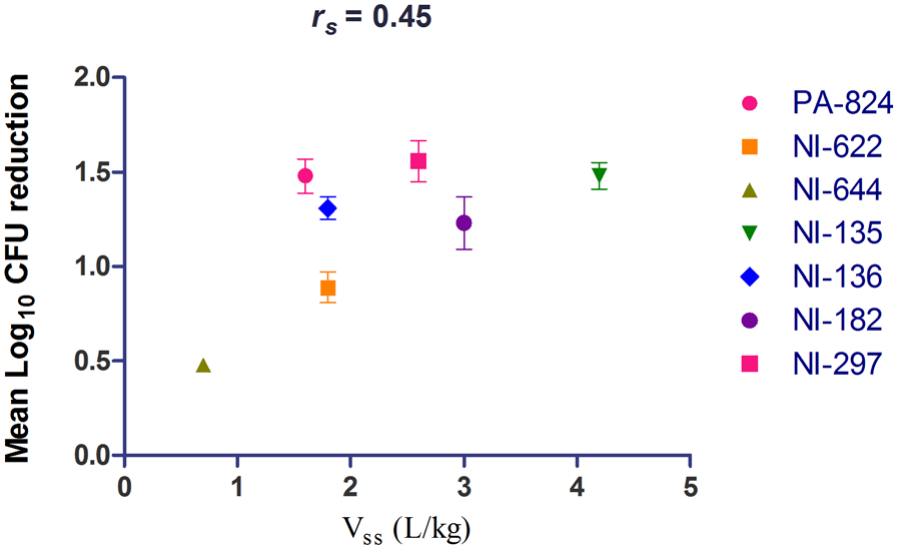
Correlation of volume of distribution with *in vivo* efficacy in mice for bicyclic 4-nitroimidazole analogs. Each data point represents Δ Mean log lung CFU reduction compared to untreated controls (mean value ± SEM from 5 animals). *r_s_* is the Spearman’s rank correlations coefficient.

Mtb mainly resides in lung granulomatous structures and hence it is important for a drug to be available at the site of the infection for it to be active. The volume of distribution is a primary PK parameter defined by the physico-chemical properties of the compound that indicates the extent of compound distribution in the body. Azithromycin, a macrolide antibiotic, with very high Vss (33 L/kg) is known to have higher lung concentration than serum (AUC lung/serum = 21) and it correlates well with *in* *vivo* activity against respiratory pathogens [44], [45]. Likewise, moxifloxacin displays a high volume of distribution (Vss = 2 to 5 L/kg) resulting in pronounced penetration into tissues (AUC L/P ratio of 3.3) [46], [47] possibly leading to its potent *in* *vivo* efficacy against TB [48]. Recently, moxifloxacin has been shown to penetrate and accumulate in granulomatous lesions in TB infected rabbit lungs [49]. TMC207, a diarylquinoline analog, extensively distributes to lungs (AUC L/P ratio of 22) and is efficacious against Mtb [50]. In this study, NI analogs having moderate-to-high volume of distribution (Vss = 1.6 to 4.2 L/kg) and L/P ratio of >2 showed good efficacy in a murine TB model (Δlog CFUs ranging from 1.23 to 1.56) (Table 2, 3 and 4, Figure 4). Interestingly, NI-622 and NI-644 that showed lower lung to plasma ratio displayed only a marginal efficacy (Δlog CFUs ranging from 0.48 to 0.89). Although, NI-135 and NI-136 showed higher lung to plasma ratio (3.6 to 4.6), their absolute lung concentrations were 2.5 to 7.5 fold lower than PA-824. However, both these compounds displayed 10 times better *in* *vitro* potency resulting in comparable *in* *vivo* efficacy to PA-824. Overall, the relationship between *in* *vivo* efficacy of bicyclic 4-nitroimidazoles displayed positive correlation with Vss (*r_s_* = 0.45) (Figure 4). Based on these observations, the Vss and lung distribution could give an initial indication about a compound’s potential for *in* *vivo* efficacy and thus these two parameters could be used for initial prioritization of compounds during early drug discovery.

A thorough dose fractionation study of PA-824 in a murine model showed that the primary PK-PD driver for *in* *vivo* efficacy is the duration during which the free concentration are above MIC (*fT_>MIC_*) in plasma [11]. In this study lung PK parameters have not been measured. Further, *fT_>MIC_* in plasma of 22%, 48% and 77% is required for it to show bacteriostatic, 1-log_10_ and 1.59 log_10_ kill respectively. In general, the PK-PD parameter driving efficacy is conserved within a given class of compounds [51], for example, the efficacy of all FQ analogs is driven by AUC/MIC [37], [52], [53], while the efficacy of β-lactams correlates with *T_>MIC_* [53]–[55]. These studies are done with thorough dose fractionation of single compound with multiple doses and dosing regimen. During lead optimization program prioritization of promising compounds that show good *in* *vivo* efficacy is important to reduce the overall turnaround time. In this retrospective analysis with 7 different bicyclic 4-nitroimidazole analogs, we attempted to correlate *in* *vivo* efficacy at 25 mg/kg with PK parameters. On the contrary to what has been observed by Ahmad et al., in this study, with seven bicyclic 4-nitroimidazole analogs having varied lung distribution, *in* *vivo* efficacy showed weak correlation with free *T_>MIC_* in plasma. However, the total *T_>MIC_* in lungs showed positive correlation with *in* *vivo* efficacy (*r_s_* = 0.88) likely due to their preferential distribution into lungs for some analogs. For all the compounds analyzed, the total lung *T_>MIC_* ranged between 64–100% resulting in 0.9–1.56 log lung CFU reduction, hence efficacy studies at lower doses (resulting in *T_>MIC_* less than 65%) might be necessary to see a better correlation. Overall, in this study a diverse set of bicyclic 4-nitroimidazoles with Vss ranging from 0.7 L/kg to 4.2 L/kg, lung to plasma ratio ranging from 0.5 to 4.6 showed positive correlation with lung *T_>MIC_* than with any other parameters.

The results presented in this study must be interpreted with a couple of limitations in mind. First, single-dose PK parameters determined in healthy mice were assumed to be similar to multiple-dose PK parameters in infected animals and were correlated with efficacy data. This assumption is supported by published preclinical data that has shown the absence of plasma accumulation of PA-824 in mice dosed for 2 months [56]. Further, in clinical studies with PA-824, the PK parameters from a single dose phase I study were similar to a multiple dose phase II study in patients [9], [10], [12], [13]. Another limitation of this study is that total concentrations in lungs rather than the free lung concentrations were used for the PK-PD analysis. It is well accepted that for a given compound unbound drug concentrations in plasma are equivalent to unbound tissue concentrations when active transport is not involved in the drug distribution [57], [58]. Further, it is the unbound concentration of a compound at its target site driving the pharmacological effect [58]–[63]. Nevertheless, whole-tissue concentrations can be of some value in early drug discovery providing a first assessment of partition into the lungs [61]. Techniques like microdialysis in lungs can be applied to assess unbound tissue concentration [64], [65]. In TB patients, Mtb mainly resides in diverse and heterogeneous lesions in lungs. In general, interpretation of PD activity of anti-TB compounds is complicated by differential lung pathophysiology. PK in intrapulmonary compartments like the epithelial lining fluid and alveolar macrophages have also been studied in humans for standard TB drugs like rifampicin [66], isoniazid [67], ethambutol [68], pyrazinamide [69], rifapentine [70], moxifloxacin [71], ofloxacin [72] and linezolid [73]. The concentration in these sites could be the key factor governing the efficacy of anti-TB drugs. However, measurement of compound concentration in lungs by microdialysis, epithelial lining fluid and alveolar macrophages have limitations in sampling, methodology and interpretation of results [61], [74]; and such studies have not been explored in preclinical settings for TB. The total lung concentration may not be equal to the concentration in Mtb lesions, thus warranting lesion PK analysis to improve the predictive power for efficacy. Recently PK in lung lesions of mycobacterium-infected rabbits has been investigated for isoniazid, rifampicin, pyrazinamide and moxifloxacin [49], [75]. Although lesion PK can offer better insights in understanding PK-PD relationships, it is not easily applicable to early drug discovery especially with mouse efficacy model as it doesn’t display spectrum of lesions observed in TB patients or in higher animal models. In addition, similar studies with bicyclic 4-nitroimidazoles may be challenging as they undergo enzymatic transformation in Mtb to multiple stable and unstable metabolites [43].

Our findings show that the efficacy of all bicyclic 4-nitroimidazole analogs is most likely driven by PK parameters in lungs. A simple efficacy surrogate would be useful during the lead optimization to prioritize candidates for lengthy efficacy studies. For this class, efficacy correlated better with concentration in lungs rather than in plasma, consistent with Vss and differential lung: plasma distributions. The results of this analysis potentially be exploited to prioritize new analogs for efficacy studies based on *in* *vitro* potency, volume of distribution and lung concentration.

## Supporting Information

File S1 Figure S1: Plasma concentration time profile for representative bicyclic 4-nitroimidazole analogs following a single 25 mg/kg oral dose in mice. Table S1: Correlation of PK parameters with *in* *vivo* efficacy in mice for bicyclic 4-nitroimidazole analogs. Table S2: Correlation of PK-PD indices with *in* *vivo* efficacy in mice for bicyclic 4-nitroimidazole analogs.
(DOCX)
