# Supplementary material for: Pharmacokinetics-Pharmacodynamics Analysis of Bicyclic 4-Nitroimidazole Analogs in a Murine Model of Tuberculosis
Source: PLoS One. 2014 Aug 20;9(8):e105222. doi: 10.1371/journal.pone.0105222 (PMC4139342; doi:10.1371/journal.pone.0105222)
Supplement: File S1 — Figure S1: Plasma concentration time profile for representative bicyclic 4-nitroimidazole analogs following a single 25 mg/kg oral dose in mice. Table S1: Correlation of PK parameters with in vivo efficacy in mice for bicyclic 4-nitroimidazole analogs. Table S2: Correlation of PK-PD indices with in vivo efficacy in mice for bicyclic 4-nitroimidazole analogs. (DOCX) [file pone.0105222.s001.docx]

**Pharmacokinetics-pharmacodynamics analysis of bicyclic 4-nitroimidazole analogs in a murine model of tuberculosis.**

Suresh B. Lakshminarayana^1#^, Helena I. M. Boshoff^2^, Joseph Cherian^1,a^, Sindhu Ravindran^1,b^, Anne Goh^1^, Jan Jiricek^1^, Mahesh Nanjundappa^1^, Amit Nayyar^2,c^, Meera Gurumurthy^1,d^, Ramandeep Singh^2, e^, Thomas Dick^1,d^, Francesca Blasco^1^, Clifton E. Barry III^2^, Paul C. Ho^3^ and Ujjini H. Manjunatha^1#^

^1^Novartis Institute for Tropical Diseases, Singapore, Singapore

^2^Tuberculosis Research Section, Laboratory of Clinical Infectious Diseases, National Institute of Allergy and Infectious Diseases, National Institutes of Health, Bethesda, Maryland, United States of America

^3^Department of Pharmacy, National University of Singapore, Singapore, Singapore

^#^ Corresponding Authors SBL & UHM

Suresh B. Lakshminarayana

Novartis Institute for Tropical Diseases, 10 Biopolis Road, #05-01 Chromos

Singapore, 138670

Email: [suresh.b_lakshminarayana@novartis.com](mailto:suresh.b_lakshminarayana@novartis.com); Phone: +65 67222991

Ujjini H. Manjunatha

Novartis Institute for Tropical Diseases, 10 Biopolis Road, #05-01 Chromos,

Singapore, 138670

Email: [manjunatha.ujjini@novartis.com](mailto:manjunatha.ujjini@novartis.com) ; Phone: +65 67222976

Key words: Tuberculosis, Pharmacokinetics, Pharmacodynamics, PA-824, Nitroimidazoles

Present address:

^a^Experimental Therapeutics Centre, Singapore

^b^National University Health System, Singapore

^c^AMRI Singapore Research Center, Singapore

^d^ Department of Microbiology, Yong Loo Lin School of Medicine, National University Health system, National University of Singapore, Singapore

^e^Translational Health Science and Technology Institute, Gurgaon, Haryana, India

**Table S1.** Correlation of PK parameters with *in vivo* efficacy in mice for bicyclic 4-nitroimidazole analogs.

| Compound ID | Dose (mg/kg) | Plasma PK | | | | Lung PK | | Mean log lung CFU reduction ± SEM |
| --- | --- | --- | --- | --- | --- | --- | --- | --- |
|  |  | Total concentration | | Free concentration | | Total concentration | |  |
|  |  | C_max_ (µg/mL) | AUC (µg.h/mL) | *f*C_max_ (µg/mL) | *f*AUC (µg.h/mL) | C_max_ (µg/g) | AUC (µg.h/g) |  |
| PA-824 | 25 | 6 | 50.9 | 0.6 | 5.1 | 17.8 | 139.9 | 1.48 ± 0.09 |
| NI-622 | 25^#^ | 7.4 | 54.2 | 0.2 | 1.6 | 5.1 | 35.6 | 0.89 ± 0.08 |
| NI-644 | 25^#^ | 8.1 | 44.8 | 0.8 | 4.5 | 3.8 | 19 | 0.48 ± 0.03 |
| NI-135 | 25 | 1.2 | 4.8 | 0.1 | 0.4 | 5.5 | 18.6 | 1.48 ± 0.07 |
| NI-136 | 25 | 2 | 10.7 | 0.3 | 1.8 | 7.2 | 39.8 | 1.31 ± 0.06 |
| NI-182 | 25 | 3.5 | 22.5 | 0.3 | 2.0 | 11.4 | 73.2 | 1.23 ± 0.14 |
| NI-297 | 25 | 6 | 99.1 | 0.1 | 1.8 | 16.3 | 233.4 | 1.56 ± 0.11 |
| Spearman correlation with efficacy |  | *r_s_* = -0.52 | *r_s_* = 0.11 | *r_s_* = -0.58 | *r_s_* = -0.16 | *r_s_* = 0.76 | *r_s_* = 0.52 |  |

PK parameters obtained from single dose PK data. Free concentrations in plasma were calculated using *in vitro* plasma protein binding. ^#^PK parameters derived from 50 mg/kg. *r_s_* = Spearman correlation coefficient. ∆ Mean log lung CFU reduction compared to untreated controls. Each data represents mean value ± SEM from 5 animals.

**Table S2.** Correlation of PK-PD indices with *in vivo* efficacy in mice for bicyclic 4-nitroimidazole analogs.

| Compound ID | Dose (mg/kg) | Plasma PK-PD indices | | | | | | Lung PK-PD indices | | | Mean log lung CFU reduction ± SEM |
| --- | --- | --- | --- | --- | --- | --- | --- | --- | --- | --- | --- |
|  |  | Total concentration | | | Free concentration | | | Total concentration | | |  |
|  |  | C_max_/MIC | AUC/MIC | *%T_>MIC_* | *f*C_max_/MIC | *f*AUC/MIC | *fT_>MIC_* | C_max_/MIC | AUC/MIC | *%T_>MIC_* |  |
| PA-824 | 25 | 20 | 170 | 66 | 2.0 | 17 | 37 | 59 | 466 | 96 | 1.48 ± 0.09 |
| NI-622 | 25^#^ | 41 | 301 | 65 | 1.1 | 8.9 | 17 | 28 | 198 | 64 | 0.89 ± 0.08 |
| NI-644 | 25^#^ | 90 | 498 | 93 | 8.9 | 50 | 53 | 42 | 211 | 84 | 0.48 ± 0.03 |
| NI-135 | 25 | 40 | 160 | 66 | 3.3 | 13.3 | 16 | 183 | 620 | 95 | 1.48 ± 0.07 |
| NI-136 | 25 | 67 | 357 | 65 | 10 | 60 | 49 | 240 | 1327 | 89 | 1.31 ± 0.06 |
| NI-182 | 25 | 175 | 1125 | 66 | 15 | 100 | 61 | 570 | 3660 | 75 | 1.23 ± 0.14 |
| NI-297 | 25 | 300 | 4955 | 100 | 5.0 | 90 | 98 | 815 | 11670 | 100 | 1.56 ± 0.11 |
| Spearman correlation with efficacy |  | *r_s_* = -0.05 | *r_s_* = -0.05 | *r_s_* = 0.30 | *r_s_* = -0.16 | *r_s_* = 0.16 | *r_s_* = 0.07 | *r_s_* = 0.63 | *r_s_* = 0.63 | *r_s_* = 0.88 |  |

PK-PD indices were calculated using PK parameters obtained from single dose PK data and *in vitro* potency. Free concentrations in plasma were calculated using *in vitro* plasma protein binding. ^#^PK parameters derived from 50 mg/kg. *r_s_* = Spearman correlation coefficient . ∆ Mean log lung CFU reduction compared to untreated controls. Each data represents mean value ± SEM from 5 animals.

**Figure S1:** Plasma concentration time profiles of representative bicyclic 4-nitroimidazole analogs following a single 25 mg/kg dose in mice
